# Recent Advances in Magnetic Polymer Composites for BioMEMS: A Review

**DOI:** 10.3390/ma16103802

**Published:** 2023-05-17

**Authors:** Zhengwei Liao, Oualid Zoumhani, Clementine M. Boutry

**Affiliations:** Department of Microelectronics, Delft University of Technology, 2628 CD Delft, The Netherlands

**Keywords:** magnetic polymer composites, micro-electro-mechanical systems, electromagnetic MEMS, bioMEMS, self-healing, shape-memory, biodegradable, microactuators, micropumps, miniaturized drug delivery systems, microvalves, micromixers, sensors

## Abstract

The objective of this review is to investigate the potential of functionalized magnetic polymer composites for use in electromagnetic micro-electro-mechanical systems (MEMS) for biomedical applications. The properties that make magnetic polymer composites particularly interesting for application in the biomedical field are their biocompatibility, their adjustable mechanical, chemical, and magnetic properties, as well as their manufacturing versatility, e.g., by 3D printing or by integration in cleanroom microfabrication processes, which makes them accessible for large-scale production to reach the general public. The review first examines recent advancements in magnetic polymer composites that possess unique features such as self-healing capabilities, shape-memory, and biodegradability. This analysis includes an exploration of the materials and fabrication processes involved in the production of these composites, as well as their potential applications. Subsequently, the review focuses on electromagnetic MEMS for biomedical applications (bioMEMS), including microactuators, micropumps, miniaturized drug delivery systems, microvalves, micromixers, and sensors. The analysis encompasses an examination of the materials and manufacturing processes involved and the specific fields of application for each of these biomedical MEMS devices. Finally, the review discusses missed opportunities and possible synergies in the development of next-generation composite materials and bioMEMS sensors and actuators based on magnetic polymer composites.

## 1. Introduction

In recent years, significant advancements have been made in the research on novel magnetic polymer composite materials [1]. These new materials, possessing properties resembling those found in nature, have opened up new opportunities, particularly in the field of biomedical research. Magnetic polymer composites offer the advantage of a polymeric matrix, which can exhibit a broad spectrum of mechanical properties. These properties range from materials with a high modulus above tens of GPa, reinforced with fibers, to elastomeric materials and hydrogels that mimic the softness of soft body organs with a Young’s modulus in the order of kPa. The polymer matrix can either be hydrophobic or hydrophilic and can either swell or remain mechanically stable in aqueous solutions or physiological conditions. They exhibit a wide range of elastic properties, from highly deformable and stretchable elastomers to rigid polymers that can be used as resonators in mechanical components of micro-electro-mechanical systems (MEMS), to wax-based composites, which are highly plastically deformable [2].

The magnetic particles used in the latest generation of magnetic polymer composites are generally either (1) powdered neodymium–iron–boron magnet (NdFeB) particles; (2) iron oxide (Fe_3_O_4_) particles with diameters varying from nanometers to micrometers, having a range of magnetic profiles from superparamagnetic particles to larger particles with multiple magnetic domains per particle; or (3) carbonyl iron (Fe(CO)_5_) particles, also referred to as iron pentacarbonyl, which are all of great interest for biomedical applications. The composite preparation process involves either (1) homogeneous dispersion of the magnetic particles within the polymer matrix, with current research efforts focusing on the mixing conditions and the use of appropriate solvents and surfactants to enhance composite homogeneity and to improve the reproducibility of magnetic and mechanical properties, or (2) the alignment of the magnetic particles along a defined axis, to anisotropically reinforce the mechanical together with the magnetic properties with the definition of a magnetic easy axis. This is accomplished by applying a homogeneous magnetic field in a predetermined direction during the polymerization or gelation process of the composite. While the mixture of polymer matrix and magnetic particles is still liquid (or has a low viscosity), the particles are mobile and can be rearranged in the composite material with an applied magnetic field, until the arrangement is frozen in a definitive version, when the polymerization process is completed. The resulting film anisotropy can be exploited in numerous applications, particularly in actuators [3].

In addition to the mechanical and magnetic properties of these composites, researchers have in recent years developed composites with other relevant properties, inspired by nature. A first property is the self-healing of materials, which is particularly important in the biomedical context [4]. While biological organisms possess the inherent capability of self-repair, this is not typically observed in man-made objects. For example, in the context of biomedical implants, which must function in the human body for extended periods, resilience against the harsh conditions posed by pH variations, immune system defense mechanisms, and enzymatic attacks is crucial. Consequently, it is necessary to devise systems that can resist and even self-repair breaches or cracks before complete failure occurs. As a result, the development of self-healing magnetic polymer composites has garnered considerable attention for the biomedical sector and beyond.

A second interesting property is shape-memory [5]. In recent years, several studies have described materials that are sensitive to both an external magnetic field and the application of heat and light, allowing a structure to be frozen in a particular shape on demand and then return to another pre-programmed shape. This property opens up possibilities in soft robotics, biomedical applications, and the development of implantable scaffolds and actuators.

A third valuable property in the biomedical context is the biodegradability of the developed composites [6]. By using composites composed entirely of biodegradable materials, an implant can be utilized for a specified duration before naturally degrading in the body, obviating the need for a secondary removal surgery and minimizing the possibility of an inflammatory response. Researchers have explored various combinations of materials with differing biodegradation time-frames, of particular interest in the development of drug delivery carriers, untethered microrobots, and the fabrication of bone scaffolds.

Current research is focused on the development of new magnetic polymer composites with the aforementioned self-healing, shape-memory, and/or biodegradability properties, as well as the development of new and ever more efficient biomedical micro-electro-mechanical systems (bioMEMS). Traditionally, MEMS systems were developed using materials and manufacturing processes that were compatible with cleanroom manufacturing of integrated circuits. These materials included silicon (Si), silicon oxide (SiO_2_), silicon nitride (SixNx), metals such as aluminum (Al), and polymers such as photoresists (e.g., SU8) and structural layers with various thermal and mechanical properties (e.g., polyimide Pi, polydimethylsiloxane PDMS). However, the development of new magnetic polymer composites has enabled the design of novel MEMS, including microactuators, micropumps, miniaturized drug delivery systems, microvalves, micromixers, and sensors, which operate through wireless magnetic actuation without requiring batteries or wiring. These new microsystems open up unexplored perspectives, particularly for diagnosis and medical monitoring with portable and implantable devices.

The objective of this review article is to provide a systematic review of the latest advances made in the last five years (January 2018 to February 2023) in the areas of (1) magnetic polymer composites and (2) biomedical electromagnetic MEMS. Indeed, research regarding novel polymer composites and cleanroom microsystem development is typically conducted by research groups with different expertise (materials scientists and chemists versus microfabrication and microelectronics experts). Therefore, the aim of this review is to identify missed opportunities and possible synergies for the development of next-generation composite materials and bioMEMS sensors and actuators based on magnetic polymer composites.

## 2. Methods

### 2.1. Literature Search

**Figure 1:**
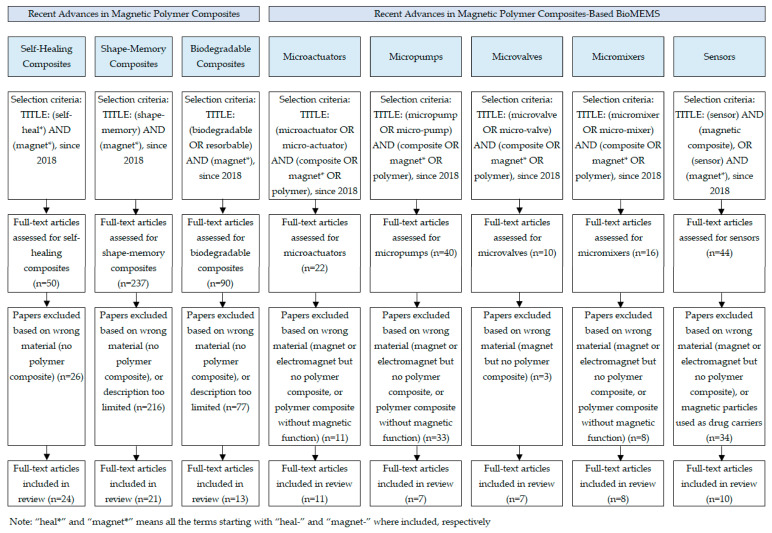
Searching strategy.

A systematic literature review was conducted using the Google Scholar electronic database (Figure 1), including only English-language journal articles and reviews. The search was limited to the period of January 2018 to February 2023, and search terms for each topic included in the review are outlined in Figure 1.

### 2.2. Evaluation Criteria

The evaluation criteria for the articles are detailed in Figure 1. The reasons for excluding certain publications are also detailed for each topic. The main reasons for exclusion from this literature search were (1) the article discussed magnets (commercially available bulk permanent magnets) or electromagnets but did not include the use of polymer composites, or (2) the article discussed polymer composites but without including magnetic properties or magnetic particles, or (3) the article focused on polymer-coated magnetic particles used as drug carriers or for in vivo medical imaging, topics outside the scope of this review article, or (4) the article focused on simulations and analytical models, or (5) the description of materials or technologies was too limited.

### 2.3. Searching Results

The subtopics discussed in this article are (1) “self-healing”, “shape-memory”, and “biodegradable composites” for the topic “recent advances in magnetic polymer composites”, and (2) “microactuators”, “micropumps”, “microvalves”, “micromixers”, and “sensors” for the topic “recent advances in magnetic polymer composites-based biomedical MEMS”. The relevant subtopics were determined based on older review articles covering magnetic polymer composites and electromagnetic MEMS and on the authors’ knowledge in the field. The number of full-text articles included in the review for each subtopic, after application of the abovementioned exclusion criteria, is shown in Figure 1. In addition, when deemed necessary by the authors, several publications were added to provide a better overview of the topic in addition to the articles selected using the terms and selection criteria in Figure 1.

## 3. Recent Advances in Magnetic Polymer Composites

Magnetic polymer composites have gained significant attention in recent years due to their potential applications, especially in the biomedical field (Table 1). Among the various aspects that have garnered interest, three stand out as exceptionally promising: self-healing composites, shape-memory composites, and biodegradable composites. Self-healing composites possess the ability to autonomously repair damage, while shape-memory composites can recover their original shape upon exposure to an external stimulus. Biodegradable composites, on the other hand, can degrade and be absorbed by the body over time. By incorporating magnetic particles into these composites, it is possible to impart magnetic properties for non-invasive monitoring and manipulation. These properties make self-healing, shape-memory, and biodegradable magnetic polymer composites attractive candidates for biomedical device development.

### 3.1. Self-Healing Magnetic Polymer Composites

Various approaches have been investigated in the development of self-healing magnetic polymer composites, including not only the choice of the matrix materials but also the magnetic particles. The assessment of self-healing composites usually centers on the duration of the healing process, the composite’s strength before and after mending, the mechanical efficacy of the repair, and the retrieval of other composite characteristics following the repair process, such as electrical conductivity. The factors which affect the effectiveness of self-healing in magnetic polymer composites are mainly related to the chemical properties (in particular the type of chemical bonds involved, their stability, and the influence of external conditions such as temperature on bond formation), the mechanical properties (including the materials Young’s modulus, the level of cross-linking, and the variation of the mechanical properties as a function of external factors such as applied temperature), and the forces applied to the core of the material itself, allowing self-healing (for example, in the case of applying a magnetic field to a magnetic polymer composite, the attractive force in the presence of a magnet leading to the closure of a crack and the healing of the material) [4,7,8,9,10,11,12,13,14,15,16].

**Figure 2:**
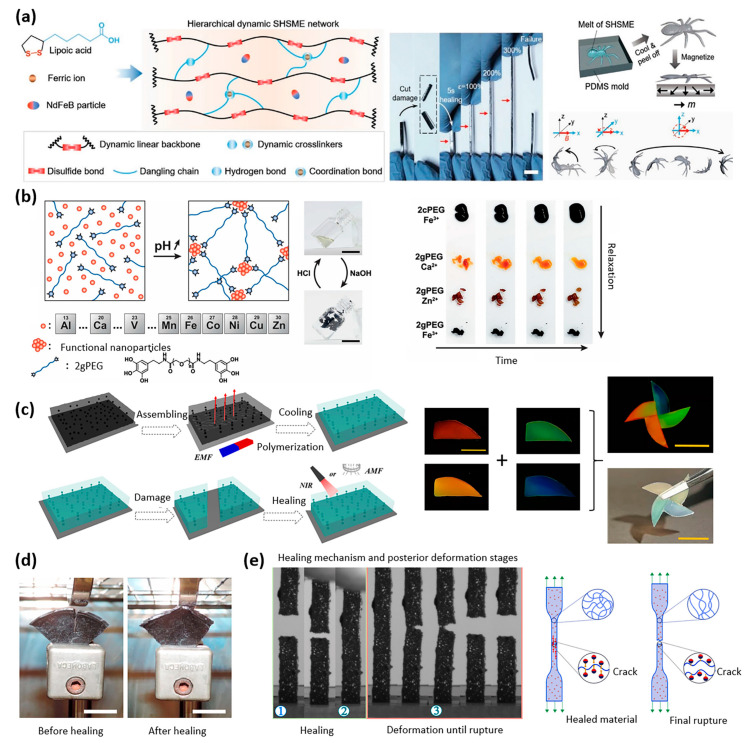
Self-healing magnetic polymer composites (1/3). (**a**) A self-healing supramolecular magnetic elastomer. Left: polymer network. Middle: cutting a composite stick (2 × 2 × 25 mm) into two pieces, self-healing for 5 s, and then stretching the healed sample till failure (scale bar, 10 mm). Right: fabrication and magnetization profile of the self-healing composite water-spider (size 10 × 6 × 1 mm), three types of locomotion (reproduced with permission from Advanced Functional Materials, Wiley) [17]. (**b**) Polyethylene glycol (PEG) functionalized with pyrogallols (2gPEG). Left: gelation mechanism of 2gPEG hydrogel. Photographs of the solution of 2gPEG and iron ions before (top) and after gelation (bottom) induced through the addition of NaOH. Right: self-healing behavior of hydrogels possessing various relaxation times (reproduced with permission from Nanoscale, RSC) [18]. (**c**) Magnetic self-healable structural color hydrogels. Left: fabrication (top) and photocontrolled healing process (bottom). Right: healing process with applied alternating magnetic field, scale bar 1 cm (reproduced with permission from ACS Appl. Mater. Interfaces, ACS) [19]. (**d**) Reversible polymer networks based on Diels–Alder thermoreversible covalent bond in a composite containing 50–100 nm Fe_3_O_4_ magnetic particles, healing experiment. An external applied magnetic field is used as the driving force to close large damage sizes. A 2 mm-thick composite sample is clamped inside an oven underneath a conventional ring magnet, with the broken gap aligned alongside the magnet. Healing temperatures in the range of 91–100 °C, close to the gel point temperature of the composite (104 °C), and for a duration of up to 1 h, resulted in healing efficiencies in the range of 56 to 100% depending on the composite’s composition, scale bar 0.5 mm. (reproduced with permission from Polymer, Elsevier) [19]. (**e**) Self-healing composite made of soft elastomeric matrix (Dowsil CY52-276) filled with hard-magnetic particles (NdFeB powder diameter of 35–55 µm), magnetized upon magnetic saturation. When approaching both broken parts, the magnetized particles interact via dipole-to-dipole interactions, closing the crack. The steps 1, 2 and 3 correspond to the sample before healing, after healing, and upon rupture (scale not specified, reproduced with permission from Composites Part B: Engineering, ScienceDirect) [20].

For soft robotics applications, the use of powders made from rare-earth permanent magnets allows for high actuation forces. The polymer matrix, on the other hand, is selected to have the necessary properties for self-healing. A millimeter-sized, high-performance, autonomously self-healing supramolecular magnetic elastomer was developed by Cheng et al. for application in soft robotics (Figure 2a) [17]. It was obtained by combining the monomer alpha lipoic acid (ALA) together with neodymium–iron–boron (NdFeB) magnetic microparticles. A water-spider soft robot with varying magnetization profiles was presented that can pick up and transport cargo when triggered by a magnetic field and was able to continue locomotion when cut-through damage was inflicted both on land and under water. Self-healing occurred based on the disulfide, hydrogen, and metal–ligand bonds derived from ALA, which is a form of intrinsic reversible healing [17].

Functionalized hydrogels are also interesting candidates for such applications: Charlet et al. developed a versatile shape-retaining and self-healing metal-coordinated hydrogel with highly tunable mechanical properties for potential use as a biocompatible medical wound sealant (Figure 2b) [18]. Polyethylene glycol (PEG) was functionalized with pyrogallol ions by means of using different crosslinkers Fe, Ca, and Zn to achieve varying strengths and self-healing times. With calcium ions, allowing fast self-healing, the hydrogel could function as an underwater adhesive, with a thin layer sustaining a pressure of 50 kPa [18]. Zhang et al. proposed another hydrogel that draws inspiration from biological systems. This hydrogel is not only biocompatible but also has the ability to self-heal due to the inclusion of magnetic nanoparticles that absorb heat. The hydrogel can be activated by either an external magnetic field or near-infrared irradiation (Figure 2c) [19]. The material was tested in vitro and was deemed suitable for cell culture and reproduction, which made this an excellent material for tissue and cell engineering. Self-healing was achieved through phase transformation by increasing the temperature to 60–65 °C [19].

Elastomers are generally interesting because of their unique mechanical properties: they are stretchable, tough, and have an adjustable Young’s modulus. As illustrated in Figure 2d, Cerdan et al. proposed using magnetostriction to self-heal large areas of damage up to 5 mm by applying a magnetic field to drive Diels–Alder’s reversible covalent bonds in the elastomer network [21]. Up to 85% healing efficiency was achieved with 10 wt% nanoparticle composites at temperatures close to 100 °C. An increase in nanoparticle concentration was shown to increase the speed of self-healing but lower the healing efficiency. This proof of concept study paves the way for promising future application in soft robotics [21].

Other sealant-type materials can also have remarkable self-healing properties. Garcia-Gonzalez et al. developed an autonomous self-healing composite that uses its sticky nature and the resulting magnetization to close cracks and tears. The healing efficiency remained identical regardless of the number of healing cycles, making this a very robust self-healing mechanism. Furthermore, this healing process was almost instantaneous, making it suitable for a dynamic environment, invaluable in the context of both bioengineering and soft robotics [20].

**Figure 3:**
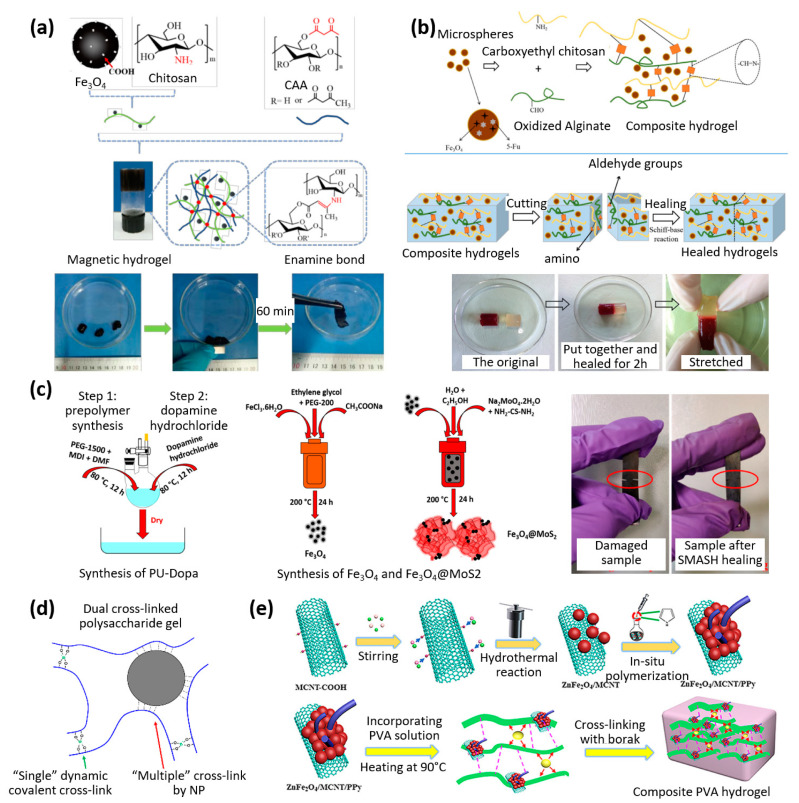
Self-healing magnetic polymer composites (2/3). (**a**) Magnetic self-healing polysaccharide hydrogel based on dynamic enamine bond. Preparation and self-healing process remotely guided by a NdFeB magnet (reproduced with permission from Chinese Journal of Chemistry, Wiley) [22]. (**b**) Carboxyethyl chitosan (CEC) and oxidized alginate (OAlg) hydrogel integrated with magnetic gelatin microspheres (MGMs) containing 5-fluorouracil (5-Fu) synthesized via Schiff-base reaction. Preparation and self-healing process (reproduced with permission from Materials Science and Engineering, Wiley) [23]. (**c**) Nanocomposite combining the shape-memory-assisted self-healing capability of “mussel-inspired” dopamine-functionalized polyurethane (PU) with synergistic electromagnetic interference-shielding capabilities from multiwalled carbon nanotubes (MWCNTs) and ferrite doped with MoS2 (Fe_3_O_4_@MoS_2_). Schematic representation of the synthesis of PU–Dopa (left) and synthesis of Fe_3_O_4_@MoS_2_ (right). The self-healing polymer was prepared by solution casting method, mixing the PU–Dopa polymer matrix with 5 wt% of Fe_3_O_4_@MoS_2_ nanoparticles and 3 wt% of carbon nanotubes MWCNTs (reproduced with permission from Applied Polymer Materials, ACS) [24]. (**d**) Carboxymethyl hydroxypropyl (CMHPG)/borate and cobalt ferrite nanoparticle self-healing composite (reproduced from Nanomaterials, Open Access) [25]. (**e**) Self-healing composite based on polypyrrole (PPy), ZnFe_2_O_4_, and multi-walled carbon nanotubes (MWCNTs) embedded in a PVA hydrogel (reproduced with permission from Journal of Alloys and Compounds, ScienceDirect) [26].

Biopolymers extracted from natural sources are also interesting because of their biocompatibility and their numerous possibilities for biomedical use. As illustrated in Figure 3a, Liu et al. synthesized a chitosan-based ferrofluid that was capable of self-healing due to the dynamic enamine bonds allowing fragments to merge into a single entity when guided by a NdFeB magnet. The ferrofluid was also able to adjust its shape to squeeze through narrow channels and has great potential for the biomedical field because of its biocompatibility and the ability to be injected directly into the target site [22].

Capable of self-healing under physiological conditions based on the Schiff-base reaction, the hydrogel developed by Chen et al. (Figure 3b) used magnetic gelatin microspheres for drug delivery and soft tissue engineering. The combination of remote control through a magnetic field and a self-healing efficiency of up to 99.1% were assumed to allow a scaffold to travel to its target location, in vivo, without being broken. Once implanted, an external magnetic field could be used to influence the drug release, and the aim was to formulate an effective platform for cancer treatment and soft tissue engineering [23].

A few matrix materials make it possible to combine several properties at the same time. Menon et al. created a polymer composite that combines dopamine-modified polyurethane, iron oxide Fe_3_O_4_, and semiconducting nanoparticles MoS_2_. This composite utilized the shape-memory effect to assist in its existing self-healing mechanism, resulting in an EMI shielding and self-healing polymer composite (as shown in Figure 3c). By incorporating carbon nanotubes (CNT), the nanocomposite was able to absorb 96% of EM waves, making it useful in flexible electronics. The tensile strength of the polymer nanocomposite was restored to 56% after self-healing, and up to 75% when CNTs were not used [24].

Zheng et al. developed a self-healing composite using thioctic acid and ferric oxide that employs three different reversible bonds (disulfide, hydrogen, and iron-carboxylate) to achieve a 100% efficiency rate within just 10 min. The inclusion of magnetic nanoparticles enhanced the strength and stability of the material, and also allowed for remote control through a magnetic field. This material showed great potential for use in shape construction, as it possesses a moldable gel property [27].

As illustrated in Figure 3d, Shibaev et al. investigated a carboxymethyl hydroxypropyl (CMHPG)/borate and cobalt ferrite nanoparticle composite that could self-heal 100% after cut-through based on its achieved elongation. Cut fragments required only 10 min in contact with each other to self-heal and could be guided with simple magnets. This gel had the potential to be used as a multi-responsive magnetically triggered actuator, and the mechanical properties were magnetically tunable. As described in Figure 3d, a dual cross-linked polysaccharide gel was obtained as follows: (1) hydrogels of CMHPG (carboxymethyl hydroxypropyl guar) were cross-linked by the addition of borate ions, which cross-linked different CMHPG macromolecules into a network due to the formation of dynamic covalent bonds with the hydroxypropyl groups of the polymer (the “Single” dynamic covalent cross-link bonds indicated in green in the Figure). (2) The addition of magnetic CoFe_2_O_4_ nanoparticles resulted in additional “Multiple” cross-link bonds (indicated in red in the Figure) [25].

The electrical properties can be successfully combined with self-repair, enabling the development of future, more robust flexible electrical circuits. Zhao et al. have developed a magnetic composite that possesses a range of unique properties, including self-healing, remoldability, high stretchability, conductivity, and electromagnetic interference (EMI) shielding (Figure 3e) [26]. This composite was created using a combination of polypyrrole (PPy), ZnFe_2_O_4_, and multi-walled carbon nanotubes (MWCNT) embedded in a PVA hydrogel, which utilized reversible boric acid ester and hydrogen bonds for self-healing. This material has significant potential for use in electronic skins, three-dimensional (3D) printing of human organs or artificial muscles, and drug delivery [26].

**Figure 4:**
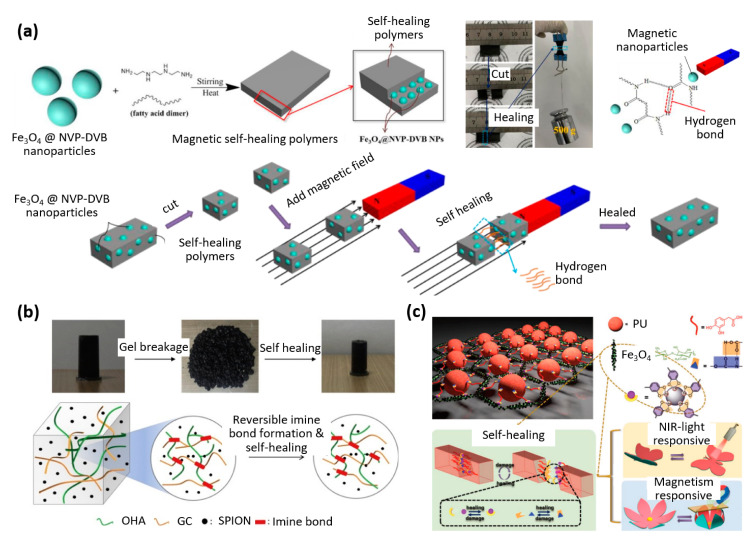
Self-healing magnetic polymer composites (3/3). (**a**) Self-healing polymer based on Fe_3_O_4_ coated with N-Vinyl-2-pyrroldone (NVP) and vivinylbenzene (DVB), and reacted with fatty acid dimer (reproduced with permission from ChemNanoMat, ACES) [28]. (**b**) Illustration of the self-healing of a ferrogel prepared from glycol chitosan, oxidized hyaluronate, and iron oxide nanoparticles. The ferrogel was fabricated using a cylindrical-shaped mold (10 mm in diameter, 10 mm in height), and the gels were ground into pieces using a mortar and pestle and placed into the mold for reassembly; after 10 min, self-healed cylinders were obtained (reproduced with permission from Carbohydrate polymers, Elsevier) [29]. (**c**) Self-healing composite made of Fe_3_O_4_/cellulose nanocrystals in a 3,4-dihydroxyphenylacetic acid (DOPAC) and polyurethane (PU) matrix (reproduced with permission from Advanced Functional Materials, Wiley) [30].

As illustrated in Figure 4a, a self-healing composite with 3-Trimethoxysilyl-propyl-methacrylate (KH570)—surface activated Fe_3_O_4_ was used by another group, Zhao et al., to coat N-Vinyl-2-pyrrolidone (NVP) and divinylbenzene (DVB) polymers [28]. The goal was to improve interface compatibility between the organic polymer and the inorganic nanoparticles. The healing efficiency of 74.3% after 1 h at room temperature increased to 78.4% when incubated and under an alternating magnetic field and can bear weights after healing based on hydrogen bonds. This material is aimed at the fields of magnetorheology, magnetostriction, and untethered robots [28].

One of the key benefits of 3D printing in manufacturing is its exceptional versatility in creating complex geometric shapes. Ko et al. utilized polysaccharides and superparamagnetic iron oxide nanoparticles to create a ferrogel that could be 3D printed using extrusion techniques (Figure 4b) [29]. This development holds promise for producing drug delivery systems and tissue engineering applications. The printed structures could be altered and fully regain their original shape after by introducing and removing a magnetic field in a matter of minutes [29].

Finally, the unique combination of self-healing capabilities and rapid actuation speed renders the following composite material exceptionally intriguing: Wang et al. developed a multi-stimuli-responsive actuator based on hierarchical structural design and interfacial supramolecular crosslinking, capable of self-healing at room temperature (Figure 3c) [30]. With a healing efficiency of 92.2% within only 15 s and able to actuate based on light and a magnetic field within 0.5 s. This is a promising material for soft robotics and biomedical microdevices and features a photothermal efficiency of 79.1% and a thermal conductivity of 31.92 Wm^−1^ K^−1^ [30].

Table 2 provides a summary of the properties, mechanism, composition, performance, and applications of self-healing magnetic polymer composites. All the self-healing mechanisms fall under the category of intrinsic reversible self-healing.

### 3.2. Shape-Memory Magnetic Polymer Composites

Shape-memory composites are a type of material that can react to external stimuli when certain conditions are met, such as electricity, light, magnetism, water, heat, or microwave radiation. Magnetic shape-memory composites are a specific type of shape-memory polymer that responds specifically to magnetic fields in addition to heat or light. These composites are widely used in various industries, including biomedical devices, artificial muscles, smart actuators, adaptive optics, and aerospace, due to their unique properties and versatility. Similar to what was discussed in the previous section regarding self-healing, the factors that affect the effectiveness of shape-memory in magnetic polymer composites are mainly related to the chemical and mechanical properties, and the influence of the applied external stimuli on the mechanical deformation of the samples. A particularly important factor is the ability of the composite material to retain a defined shape when heating/cooling and mechanical deformation steps are applied under specific conditions and in a predefined sequence, or when other stimuli such as magnetic field and light are applied. The combined study of thermal, mechanical, and magnetic properties of any new composite formulation is crucial in this context [5,31,32,33,34,35,36,37,38,39].

**Figure 5:**
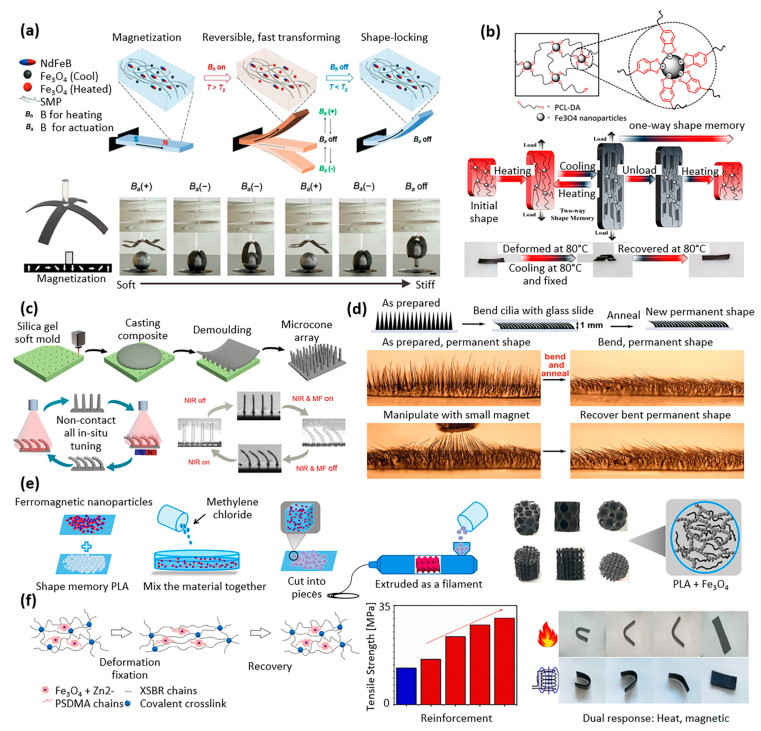
Shape-memory magnetic polymer composites. (**a**) Magnetic shape-memory composite with Fe_3_O_4_ and NdFeB magnetic particles embedded in an amorphous acrylate-based polymer matrix (reproduced with permission from Advanced Materials, Wiley) [40]. (**b**) Metallo-supramolecular poly(ε-caprolactone) (PCL)-based network using catechol chemistry, with thermal/magnetic/light-responsive two-way shape-memory effects. Illustration of the thermal-based shape-memory behavior of the composite (reproduced with permission from Macromolecules, ACS) [41]. (**c**) Shape-memory composites made of epoxy mixed with 5 μm iron particles, reversibly tuned between the tilted and upright state under near-infrared (NIR) irradiation and magnetic field (MF) actuation (reproduced with permission from Advanced Functional Materials, Wiley) [42]. (**d**) Photothermally reconfigurable magnetic cilia made of iron particles with thermoplastic polyurethane. The array of cilia was sheared between two glass slides to create the bending deformation, followed by clamping the array of bent cilia between the slides and annealing at high temperature (80 °C or 180 °C depending on the polymers used as matrix). After cooling and removing the mechanical constraints, the new permanent shape was set and persists after manipulating the cilia with a magnet (shown in the Figure). The same effect was also observed when combining a magnet with illumination to set a temporary shape of cilia and then illuminating without the magnet to recover the permanent shape (not shown in the Figure). (reproduced with permission from Advanced Materials Technologies, Wiley) [43]. (**e**) Bone tissue scaffolds made of magnetic shape-memory polymer composites, processed by 3D printing technology (reproduced with permission from Composites Science and Technology, ScienceDirect) [44]. (**f**) Shape-memory polymers based on carboxylic styrene butadiene rubber (XSBR)/ferriferrous oxide (Fe_3_O_4_)/zinc dimethacrylate (ZDMA) (reproduced with permission from Ind. Eng. Chem. Res., ACS) [45].

As illustrated in Figure 5a, Ze et al. developed a magnetic shape-memory composite which consisted of two types of magnetic particles (Fe_3_O_4_ and NdFeB) in an amorphous acrylate-based polymer matrix [40]. The matrix could be softened via magnetic inductive heating of the magnetic particles. In addition, high-remanence particles with reprogrammable magnetization profiles drove the rapid and reversible shape change under actuation magnetic fields. Once cooled, the actuated shape could be locked. This shape-memory composite was developed with the objective of being used in soft robotic applications [40].

The use of bio-based materials for biomedical applications offers the advantages of biocompatibility, biodegradability, and reduced environmental impact. In addition, benzoxazines are of particular interest owing to the abundant availability and affordability of their starting materials as well as the simplicity of their one-pot synthesis. Leungpuangkaew et al. studied a magnetic shape-memory composite composed of bio-based benzoxazine resin and Fe_3_O_4_. The composite exhibited a shape recovery effect that could be triggered by either an alternating magnetic field or exposure to light. Incorporating Fe_3_O_4_ nanoparticles into nanocomposites significantly enhanced their shape-memory performance. The addition of 3 wt% Fe_3_O_4_ nanoparticles increased the shape fixity from 85% in the neat copolymers to 93% in the filled copolymers, while the shape recovery increased from 94% to 98% [46].

Nature-inspired design can lead to the creation of advanced materials with superior properties and functionality. As illustrated in Figure 5b, Du et al. investigated a metallo-supramolecular poly(ε-caprolactone) (PCL)-based network using catechol chemistry, with thermal/magnetic/light-responsive two-way shape-memory effects (Figure 5b) [41]. The design was inspired by the adhesion strategy of mussels. The hybrid networks became self-assembled upon the metal coordination interaction between superparamagnetic iron oxide nanoparticles and catechol–telechelic PCL. The shape recovery effect could be activated through exposure to an alternative magnetic field, direct heating at 80 °C, or light exposure [41].

Li et al. investigated shape-memory composites made of epoxy mixed with 5 μm iron particles. A silica gel mold was used to form arrays of microcones (or micro-cilia) made of the shape-memory composite, and the orientation of the microcones was reversibly tuned between the tilted and upright state under near-infrared (NIR) irradiation and magnetic field (MF) actuation (Figure 5c) [42]. The unidirectional transport of liquid droplets on the tilted microcones was investigated, together with other applications including selective droplet release and reusable temperature switch [42].

As shown in Figure 5d, Liu et al. produced reconfigurable magnetic cilia by mixing iron particles with thermoplastic polyurethane (Diaplex) and performing solvent casting between two permanent magnets. The magnetic moments of the microparticles, aligned by the applied magnetic field, led to the self-assembly of magnetic particles in the cilia in the direction of the field. These resulting magnetic cilia were reconfigurable using light and magnetic fields as remote stimuli. Temporary shapes achieved through magnetic actuation and photothermal heating could be locked by turning off the light and magnetic field. Then, turning on the light without the magnetic field enabled the recovery of the permanent shape [43]. Previously, the same group demonstrated other applications of this magnetic shape-memory composite, such as the creation of cantilevers and flowers that exhibited multiple instances of locking and unlocking, bistable snappers that could be activated through magnetic or optical means, and grabbers that could repeatedly grasp and release objects [47].

The internal structure of plants can also serve as a model for the design of innovative scaffolds: as illustrated in Figure 5e, Zhao et al. developed bone tissue scaffolds made of magnetic shape-memory polymer composites, processed by 3D printing technology [44]. The geometry of these scaffolds was inspired by the microstructure of lotus root and cellular co-continuous-like structures. In addition, the incorporation of magnetic nanoparticles into the polylactic acid-based composites offered the possibility of implanting bone tissue scaffolds into the body in a contracted state and then deploying them to fit perfectly into the cavity to be filled. The scaffolds’ shape-memory effect also holds potential advantages for minimally invasive surgery. Overall, these porous scaffolds based on shape-memory composites have promising applications in bone tissue repair and regeneration [44].

Styrene-butadiene rubber is a widely used material that makes up 50% of car tires. Liu et al. reported dual-response shape-memory polymers based on carboxylic styrene-butadiene rubber (XSBR)/ferriferrous oxide (Fe_3_O_4_)/zinc dimethacrylate (ZDMA) (Figure 5f) [45]. Fe_3_O_4_ was added to provide XSBR with magnetic property and increase its glass transition temperature. Zinc dimethacrylate was used to improve the compatibility of Fe_3_O_4_ and XSBR, further improving the glass transition temperature of the composite by introducing ionic cross-linking points. The shape fixation ratio of the shape-memory polymers was 100% at room temperature, the shape recovery ratio was 100% in both a thermal field and an alternating magnetic field, and the tensile strength reached 30.26 MPa [45].

Chen et al. developed a shape-memory polymer based on biocompatible poly(ε-caprolactone) (PCL), thermoplastic polyurethane (TPU), polydopamine (PDA), and iron oxide. The nanocomposite film was light- and magnetic-responsive, taking approximately 1 s and 5 s for a cm-long sample to respond to magnetic field and light, respectively. Similarly to the other magnetic shape-memory composites with dual actuation mechanism, the combination of photothermal heating and magnetic-responsive actuation resulted in a temporary shape that could be locked by turning off the light and magnetic field. Subsequent light illumination without the magnetic field was used to drive the recovery of the initial shape [48].

**Figure 6:**
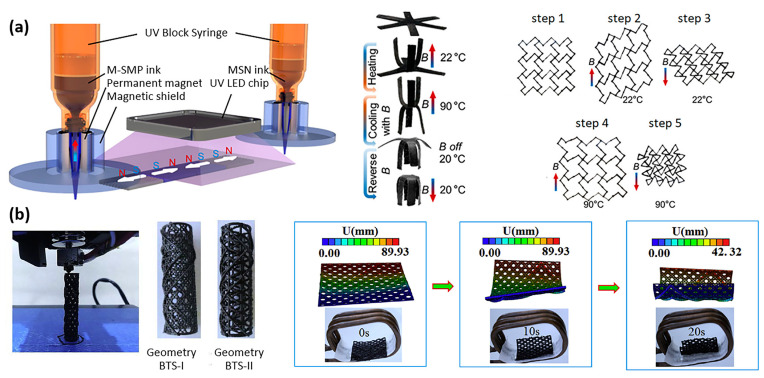
The 3D-printed shape-memory magnetic polymer composites and 3D printing of magnetic shape-memory polymers. (**a**) Multimaterial 3D printing technology for magnetic soft composites and magnetic shape-memory polymers. Left: 3D printer schematic. Middle: shape transformation. Right: active metamaterial. Initial shape (step 1), deformed shapes at 22 °C under upward (step 2) and downward (step 3) magnetic fields. Deformed shapes at 90 °C under upward (step 4) and downward (step 5) magnetic fields (reproduced with permission from Applied Materials & Interfaces, ACS) [49]. (**b**) The 3D printing of magnetic shape-memory polymer composites made of polylactic acid (PLA) and Fe_3_O_4_, to fabricate personalized tracheal scaffolds. Left: 3D printing process at 180 °C. Right: wireless actuation (reproduced with permission from Composites Science and Technology, Elsevier) [50,51].

Three-dimensional printing has numerous advantages for the production of biomedical implants, scaffolds, and actuators. Firstly, it allows for the creation of patient-specific devices that are tailored to the individual’s anatomy, leading to better treatment outcomes. Secondly, 3D printing enables the production of complex geometries and internal structures that are not feasible with traditional manufacturing methods. Lastly, 3D printing is a cost-effective and rapid prototyping technology, which can speed up the development process of biomedical devices and bring them to market more quickly. The following works have leveraged 3D printing for magnetic shape-memory polymers. As illustrated in Figure 6a, Ma et al. developed a multimaterial 3D printing technology for magnetic soft composites and magnetic shape-memory polymers, with simultaneous fine control of temperature and applied magnetic field, to explore their tunable properties. Using both thermal and magnetic actuation, they demonstrated multiple deformation modes with unique shape configurations, synthetizing, e.g., active metamaterials with tunable physical properties such as sign-change Poisson’s ratio [49]. Zhao et al. developed magnetic shape-memory polymer composites dedicated to the personalized 3D printing of a bio-designed tracheal scaffold (Figure 6b) [50]. The authors utilized the term “4D printing” to describe the use of specific properties of intelligent materials, such as shape-memory polymers, in their printing process. To prepare for 3D printing, a composite consisting of biodegradable polylactic acid (PLA) and Fe_3_O_4_ particles was poured into a twin-screw extruder to obtain a filament. The filament was then heated to approximately 180 °C, the melting temperature of PLA, in order to print the scaffolds [50]. Zhang et al. investigated the use of the same material as an injectable and expandable filler for bone repair [51].

Table 3 presents a summary of the properties of shape-memory polymer magnetic composites described in the articles that met the selection criteria and are discussed in this review.

### 3.3. Biodegradable Magnetic Polymer Composites

The development of biodegradable magnetic composites typically involves the combination of biodegradable polymers or hydrogels with iron oxide particles. Biodegradable polymers are a class of materials that can be broken down into simpler, harmless compounds through biological processes. Some examples of biodegradable polymers include poly(lactic acid) (PLA), poly(glycolic acid) (PGA), poly(ε-caprolactone) (PCL), polysaccharides such as chitosan and alginate, polyhydroxyalkanoates (PHAs), and poly(trimethylene carbonate) (PTMC) [6,52,53,54].

The porous bone tissue scaffolds developed by Zhao et al. (Figure 5e) [44] and based on magnetic shape-memory polymer composites are made of PLA and Fe_3_O_4_ particle. PLA is a biodegradable polyester degrading into lactic acid. The Food and Drug Administration (FDA) has approved PLA for specific applications within the human body, such as a drug delivery device and tissue engineering [55]. In [44], the scaffolds’ biocompatibility was evaluated in vitro, resulting in a proliferation of the osteoblasts and excellent cell adhesion similar to control cultures. It was observed that smaller pores corresponded to better cell adhesion, while larger pores were more beneficial for the exchange of nutrients, important in the context of long-term implantation [44].

The 3D-printed personalized tracheal scaffolds presented earlier by Zhao et al. (Figure 6b) were also made from the same biodegradable materials (PLA and Fe_3_O_4_) [50]. Biodegradable tracheal scaffolds are highly valuable due to their ability to offer mechanical reinforcement to the tracheal wall during the early post-surgery phase. As the cell tissue develops and integrates with the scaffold structure, it can gradually break down and be naturally eliminated, preventing the need for a second surgical intervention and the associated risk of further injury [50]. For the same reasons, Zhang et al. studied this composite for bone repair [51].

The magnetic polymer composites developed by Chen et al. based on poly(ε-caprolactone) (PCL), are also biodegradable [48]. Indeed, PCL is a biodegradable polyester that exhibits a low melting point of approximately 60 °C and a glass transition temperature of −60 °C. Due to its ability to break down through hydrolysis of its ester linkages in physiological environments, PCL has garnered significant interest as a potential implantable biomaterial. It is FDA-approved for application as a drug delivery device, suture, or adhesion barrier [56]. In general, the combination of biodegradability and shape-memory properties in composites is very promising in the field of biomedical devices, artificial muscles, and smart actuators.

**Figure 7:**
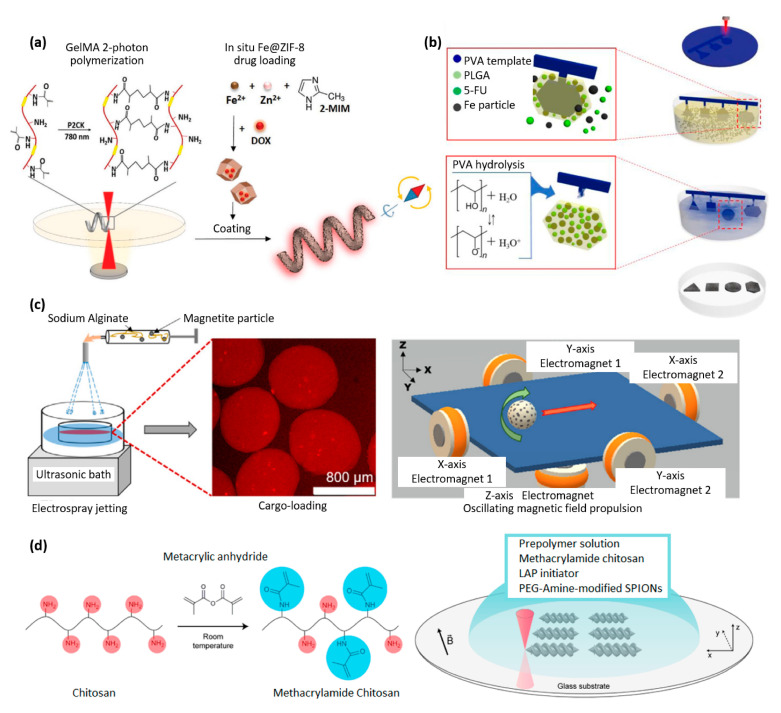
Biodegradable magnetic polymer composites. (**a**) Biodegradable helical microrobot, made of gelatin methacryloyl and fabricated by two-photon polymerization (reproduced with permission from Advanced Healthcare Materials, Wiley) [57]. (**b**) Biodegradable PLGA/Fe/5-FU microrobots, fabrication process (reproduced with permission from Sensors and Actuators B: Chemical, ScienceDirect) [59]. (**c**) Biodegradable capsules for drug delivery made of alginate hydrogel, loaded with Fe_3_O_4_ particles (reproduced with permission from Journal of Colloid and Interface Science, Elsevier) [60]. (**d**) Biodegradable microswimmers for drug delivery based on chitosan loaded with iron oxide (magnetite) particles (reproduced with permission from ACS Nano) [61].

The rapid degradation of gelatin compared to PLA and PCL is an advantage for many applications. Terzopoulou et al. developed a biodegradable gelatin-based helical microrobot that is able to realize magnetic locomotion, drug delivery, and selective degradation in cell cultures (Figure 7a) [57]. The chassis of the robot consisted of biodegradable hydrogel microstructures made by direct-laser writing of gelatin methacryloyl using two-photon polymerization. Gelatin methacryloyl degrades via enzymatic hydrolysis, and the complete degradation of the microrobot is observed after two weeks [57]. Dong et al., from the same group, developed soft magnetoelectric microswimmers for drug delivery, also printed by two-photon polymerization and loaded with a drug for the treatment of neurodegenerative diseases [58].

As illustrated in Figure 7b, Kim et al. also worked on biodegradable magnetic polymer composites and biodegradable microrobots for drug delivery that were made of three key materials: poly(DL-lactic-co-glycolic acid) (PLGA) for biodegradability, iron (Fe) particles for magnetic properties, and 5-fluorouracil (5-FU) as the drug material [62]. The fabrication procedure for PLGA/Fe/5-FU microrobots included (1) the UV-laser micro-machine cutting of a poly(vinyl alcohol) PVA sheet into the various shapes to form the base of the microrobots; (2) the formation of the PLGA/Fe microrobot on the PVA template whilst simultaneously encapsulating the 5-FU; (3) the removal of the PVA template by dissolution [59]. The same group developed a microrobot with a 3D helical structure made of poly(ethylene glycol) diacrylate (PEGDA), pentaerythritol triacrylate (PETA), Fe_3_O_4_ nanoparticles, and 5-fluorouracil (5-FU) [63].

Calcium alginate is another interesting biodegradable material usually used in the fabrication of wound dressings due to its excellent water absorption capacity, biocompatibility and film-forming properties. Rutkowski et al. took advantage of it in the context of the fabrication of biodegradable capsules for drug delivery (Figure 7c) [60]. The composite consisted of a calcium alginate hydrogel, loaded with Fe_3_O_4_ particles, and the capsules were fabricated using hydrodynamic electrospray ionization jetting. The capsules’ size could be adjusted within the range of 10 μm to 2 mm, and they were utilized for encapsulating a model drug. The magnetic particles allowed for controlled directional motion under a rotating magnetic field. In addition, ultrasound actuation was used for on-demand drug release [60].

Chitosan is a natural polysaccharide derived from the chitin found in the exoskeletons of crustaceans, such as crab, lobster, and shrimp. As shown in Figure 7d, Bozuyuk et al. produced microswimmers for drug delivery through two-photon polymerization using chitosan combined with iron oxide particles. The resulting product was a magnetic polymer composite that was biodegradable and suitable for use in a biological environment. The drug release mechanism of the microswimmers was controlled through a photocleavage-based light-triggered delivery approach [61].

Table 4 provides an overview of the properties of biodegradable magnetic polymer composites discussed in the present review.

## 4. Recent Advances in Magnetic Polymer Composite-Based Biomedical MEMS

Biomedical micro-electro-mechanical systems (BioMEMS) are devices that integrate mechanical and electrical components with biological materials to perform specific biomedical functions. The pacemaker and cochlear implant are well-known examples of BioMEMS that have been successfully used to treat cardiovascular and auditory disorders, respectively. Other examples include microfluidic devices for drug delivery and diagnostics, microsensors for monitoring glucose levels in diabetes patients, and microelectrodes for neural stimulation and recording in brain–machine interfaces [64]. Magnetic polymer composites allow for the development of a new generation of MEMS with wireless electromagnetic control, as described hereafter.

### 4.1. Magnetic Polymer Composite-Based Microactuators

Magnetically controlled soft actuators using magnetic-responsive composites have gained significant attention due to their straightforward fabrication process and the flexibility of the control strategies employed [65,66].

**Figure 8:**
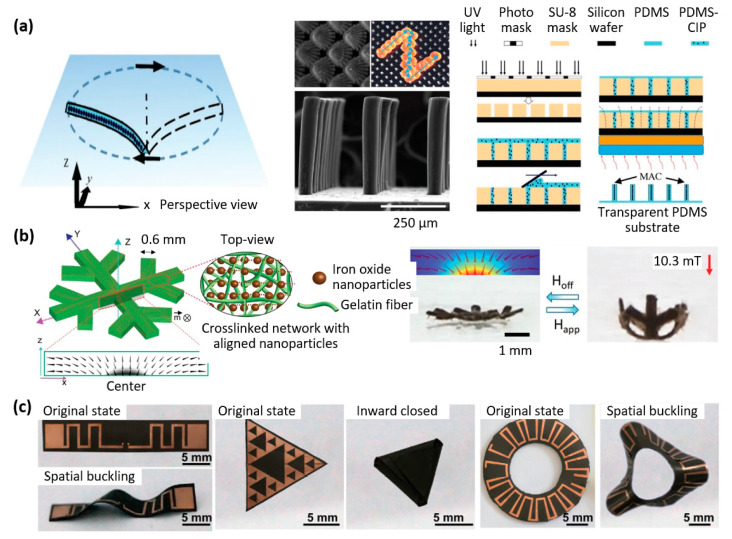
Magnetic polymer composite-based microactuators (1/2). (**a**) Micro-molded magnetic artificial cilia made of carbonyl iron particles in PDMS matrix. Left: actuation principle. Center: scanning electron microscopy images of the cilia. Right: fabrication process (reproduced with permission from Advanced Functional Materials, Wiley) [67,68]. (**b**) Biodegradable magnetic hydrogel milli-gripper. Left: schematic representation of the inner structure of the microactuator with directionally self-assembled iron-oxide particle chains. Right: bending of the micro-gripper when a magnetic field is applied (reproduced with permission from Advanced Functional Materials, Wiley) [69]. (**c**) Magnetic composite-based origami membranes used as soft actuators and carriers for flexible electronics (reproduced with permission from Advanced Materials Technologies, Wiley) [70].

Zhang et al. developed a microactuator for removal of microparticles based on the design of a ciliated surface (Figure 8a). Biological cilia are hair-like structures that are highly effective in manipulating particles for a variety of purposes, such as feeding, preventing fouling, and transporting cells. Drawing inspiration from the adaptability of cilia, the researchers successfully demonstrated the ability of self-cleaning surfaces covered with micro-molded magnetic artificial cilia to actively remove particles [67,68].

Beyond microactuators, examples of larger actuators are interesting, as the proposed mechanisms are also exportable to the micrometer scale and propose an interesting approach for the design of future microsystems. Taking advantage of the orientability of magnetic particles in a composite polymer when exposed to a magnetic field during polymer curing, Goudu et al. [69] also developed a biodegradable untethered magnetic hydrogel milli-gripper. The microactuator, illustrated in Figure 8b, consisted of a collagen-based hydrogel network with embedded superparamagnetic iron oxide nanoparticles. Its ability to undergo flexible and reversible shape deformations was demonstrated when subjected to a magnetic field in both glycerol and biologically relevant liquid media [69].

Another example of a recently developed microactuator is the flexible origami magnetic membranes by Qi et al. [70]. As shown in Figure 8c, a layer of flexible electronics was laminated on top of a magnetic polymer composite, whose programmable deformation was used to achieve reconfigurable electrical properties, controllable deployment, and tunable modes of operation. This microactuator has potential applications in surgical robots, tunable antennas, and various reconfigurable flexible electronics [70].

**Figure 9:**
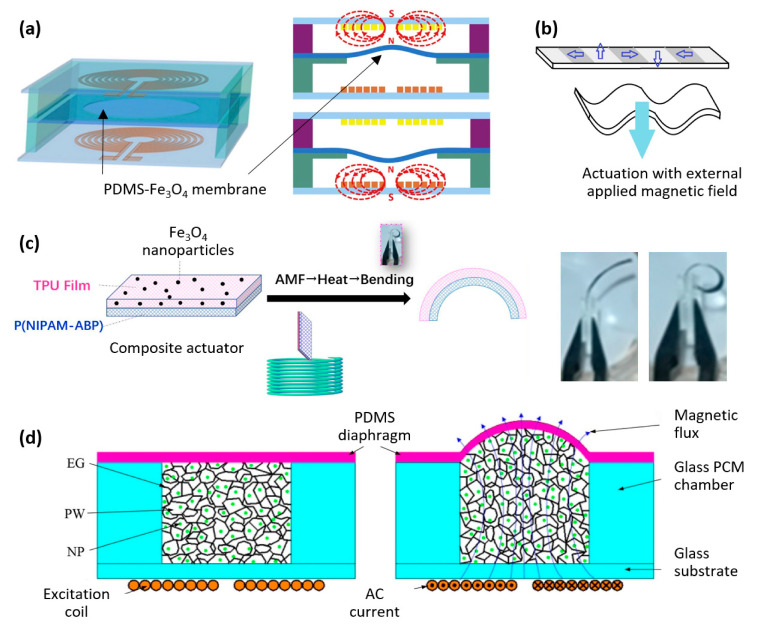
Magnetic polymer composite-based microactuators (2/3). (**a**) Bi-directional electromagnetic microactuator based on PDMS-Fe_3_O_4_ nanocomposite magnetic membrane (reproduced with permission from Microelectronic Engineering, Elsevier) [71]. (**b**) A new technique for microstructuring gel-based magnetic composites for microactuators, which relies on using a laser to soften certain areas of the composite and exposing them to magnetic fields in different directions [72]. (**c**) Alternative magnetic field-induced thermo-responsive composite actuator. AMF: Alternative magnetic field. (Reproduced with permission from Nanomaterials, MDPI) [73]. (**d**) Working principle of the phase change material-composite-based microactuator with induction heating. EG, PW, and NP stand for expanded graphite, paraffin wax, and nickel particle, respectively (reproduced with permission from Sensors and Actuators A, Elsevier) [74].

Paknahad et al. developed an electromagnetic microactuator utilizing a PDMS-Fe_3_O_4_ nanocomposite magnetic membrane, which was tested for both uni-directional and bi-directional actuation, as shown in Figure 9a [71]. Membrane deflections of 8 μm and 66 μm were achieved within 2 s and 20 s, respectively, representing an improvement of more than one order of magnitude in terms of speed compared to the state-of-the-art.

This example highlights how enhancing the response time of soft microactuators remains a significant challenge, necessitating the optimization of composite materials and design refinements. Wang et al. addressed this issue by developing a microactuator that combines rigid and flexible parts to improve both the driving force and stability. By adopting this approach, the rigid sections were assigned responsibility for quick magnetic response, while the soft areas exhibited desired deformation performance [75].

Ninomiya et al. introduced a novel technique for microstructuring gel-based magnetic composites, imparting various magnetic orientations in different regions of the material (Figure 9b) [72]. The goal of this method was to program the deformation of the microactuator. In this process, a specific orientation was imparted to magnetic particles in a gel solution by applying an external magnetic field, after which gelation occurred. Next, an IR laser was employed to heat and melt specified areas on the composite surface, under a weak magnetic field with a different orientation. The magnetic particles within the molten region aligned themselves with this applied magnetic field. After gelation of these regions, a soft magnetic composite with a fine magnetic pattern was obtained, allowing controlled deformation of the microactuator [72].

The heating effect of magnetic particles when an AC external magnetic field is applied has also recently been utilized to develop microactuators. Li Liu et al. introduced a thermo-responsive microactuator based on a bi-layer structure (Figure 9c). The device comprises a polyurethane film with embedded Fe_3_O_4_ magnetic particles, laminated on top of a polymer film (P(NIPAM-ABP)) with different thermal expansion coefficients. When subjected to an alternating magnetic field, the composite heats up, and the microactuator bends in a few seconds [73].

Bendong Liu et al. demonstrated another practical use of the heating effect of magnetic particles when exposed to an AC magnetic field. They developed a microactuator that utilized a phase change material. The composite material contained paraffin wax, graphite, and nickel particles, whose volume could be controlled by heating. The microactuator achieved 140 µm actuation height within 5 s when subjected to an input power of 1.42 W and an excitation frequency of 1 kHz (Figure 9d). Additionally, a fabrication process was developed for integrating the microactuator into MEMS [74].

### 4.2. Magnetic Polymer Composite-Based Micropumps

Micropumps have a wide range of applications in the medical field. They can, e.g., be used for precise and controlled delivery of drugs, or in microfluidic analysis, where they can manipulate and analyze small volumes of fluids, allowing for high-throughput testing and faster diagnosis of diseases. Electromagnetic micropumps generally consist of a cavity connected to an inlet and outlet, covered by a flexible, magnetically responsive membrane, the deformation of which is controlled remotely by an alternating magnetic field. A system of microvalves makes it possible to impose a pumping direction to the fluid [76,77].

Shinoda et al. [78] utilized a fabrication process similar to Zhang et al. [67,68] to create artificial cilia, with the aim of developing a micropump. One notable difference between these two studies is that, in Shinoda’s work, adjacent cilia were fabricated with different orientations of magnetic particle chains in order to mimic the metachronal wave, a characteristic motion observed in natural cilia movement of living organisms [78].

Saren et al. developed a wireless micropump made from the magnetic shape-memory alloy Ni–Mn–Ga. This material can undergo a shape change in response to an external magnetic field, which causes the fluid to move in a peristaltic-like manner. This approach eliminates the need for electrical contacts and simplifies the design by eliminating the mechanical parts typically found in traditional pumping technologies, resulting in a less complex micropump [79]. Harmel et al. also recently proposed a micropump based on a Ni–Mn–Ga shape-memory alloy actuator, with a design in which the pump is divided into disposable and non-disposable components [62].

**Figure 10:**
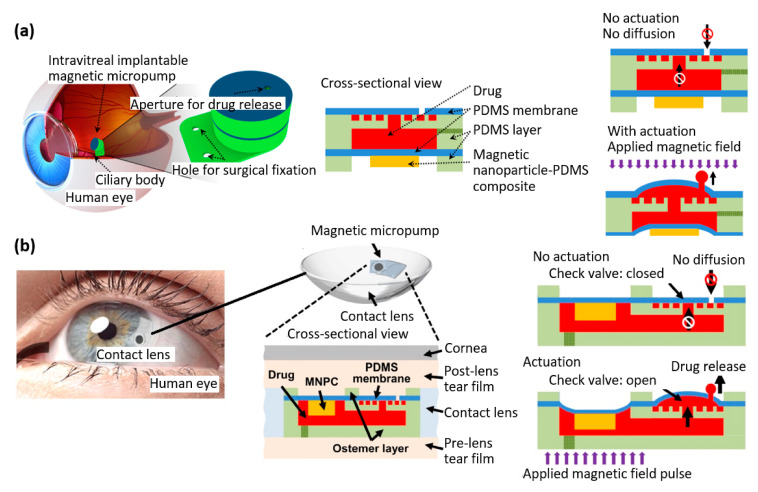
Magnetic polymer composite-based micropumps. (**a**) Intravitreal implantable magnetic micropump that included a micro check valve. Left: fixation of the magnetic micropump at the ciliary body. Center: micropump architecture. Right: actuation mechanism [80]. (**b**) Magnetic micropump embedded in contact lens for on-demand drug delivery. Left: contact lens architecture. Right: actuation principle, magnetic nanoparticle (10 nm iron oxide particles) Fe_3_O_4_-PDMS composite [81].

Wang et al. conducted a study on an intravitreal implantable magnetic micropump that included a micro check valve (Figure 10a) [80]. This system enabled targeted drug delivery to vascular endothelial growth factor receptors to treat various eye pathologies. The precise release of drugs on demand was achieved through the deflection of a magnetic membrane in response to an external magnetic field. The actuation membrane of the micropump was fabricated by assembling a cylindrical block made of magnetic composite (consisting of magnetic nanoparticles and PDMS) on top of a layer of PDMS. The final magnetic micropump was 5 × 5 mm with a thickness of 2 mm and suture tab of 5 × 7 × 0.2 mm [80].

Afterwards, the same group developed a magnetic micropump embedded in contact lenses for on-demand drug delivery (Figure 10b) [81]. The micropump they proposed can be activated using an external magnetic field without requiring a battery. They also incorporated a micro check valve into the micropump to enable one-way drug delivery from the pump to the post-lens tear film. By activating the external magnetic field, the micro check valve was opened, allowing for on-demand drug release. Conversely, when there was no external magnetic field present, the micro check valve remained closed, preventing unwanted drug diffusion. The magnetic micropump was 5 × 9 × 0.5 mm, and it was integrated into a lens made of the polymer Ostemer 322 [81].

Tahmasebipour et al. developed a unidirectional and bidirectional valveless electromagnetic micropump with a PDMS-Fe_3_O_4_ nanocomposite magnetic membrane. From a design point of view, the main difference with Wang’s work is that the actuating membrane of the micropump was made entirely of a magnetic composite. With this design, a maximum flow rate of 1.25 µL min^−1^ at the frequency of 0.1 Hz was achieved [82].

### 4.3. Magnetic Polymer Composite-Based Microvalves

Microvalves are small-scale valves used in various fields, including microfluidics and biomedical engineering, for the precise control of fluid flow. They are essential components in lab-on-a-chip devices, which enable the integration of multiple laboratory functions on a single chip. Microvalves also have applications in inkjet printing, aerosolization, and microscale actuation systems [76].

One of the earliest examples of a magnetically actuated microvalve was developed by Sadler et al. for liquid and gas control applications [83]. In this early device, a 3 mm × 5 mm × 10 μm silicon diaphragm was covered with a 2 mm × 2 mm × 7 μm electrodeposited Ni/Fe permalloy layer. The opening and closing movement of the valve was controlled by the magnetic field produced by an external coil with a 25 mm thick magnetic core. The development of soft, flexible, magnetically active polymer composites and their integration into microvalves improved actuation efficiency by reducing the energy required to move the diaphragm, while the development of less complex manufacturing processes allowed for greater batch-to-batch reproducibility, more sophisticated unit design, and an overall reduction in manufacturing costs, as illustrated by the recent work of Pradeep et al. [84].

**Figure 11:**
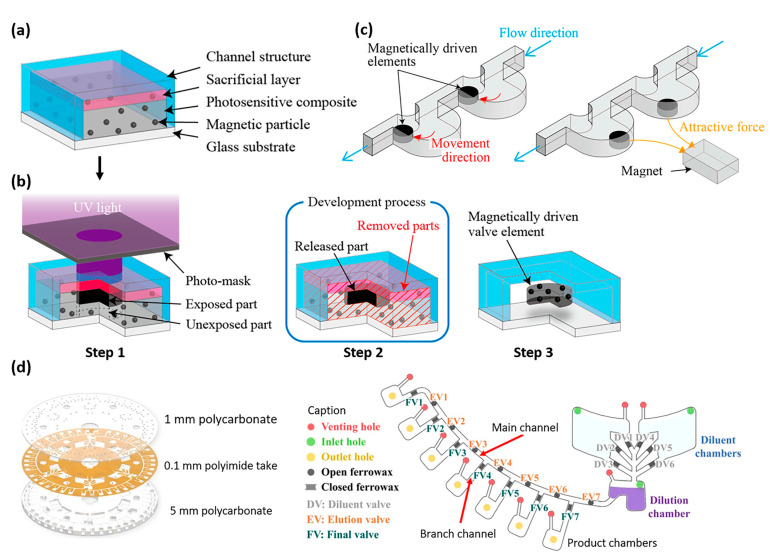
Magnetic polymer composite-based microvalves. Fabrication of magnetically driven microvalve arrays using a photosensitive composite: (**a**) materials, (**b**) fabrication process (step 1: exposure to UV light, step 2: development, step 3: released microvalve), and (**c**) operation principle of the magnetically controlled microvalves (reproduced and adapted with permission from Magnetochemistry MDPI) [85]. (**d**) Microvalves based on a ferrowax composite, for an automated diagnostic microsystem involving serial dilution processes (reproduced with permission of the authors and Authorae) [86].

Nakahara et al. recently proposed a simplified fabrication process to synthesize magnetic composites and fabricate microvalves used to control fluids in micro total analysis systems (µTAS) [85]. They developed a composite based on photoresist (SU-8 3010) and magnetic particles (pure iron particles with diameter of 3–5 µm). As shown in Figure 11a,b, the fabrication process consisted of (1) fabricating a microfluidic chip with transparent microchannels (glass and micromolded PDMS); (2) coating the inside of the microchannel with a sacrificial layer (AZP4620 photoresist); (3) injecting the magnetic polymer composite in the microchannel; (4) only exposing certain areas of the composite to UV with a shadow mask; and (5) removing the uncured composite and the sacrificial layer, resulting in the magnetic actuator being embedded in a microchannel, as illustrated in Figure 11c.

Alam et al. studied a bidirectional electromagnetic microvalve for drug delivery with two equidistant microcoils placed on either side of a flexible polymer membrane (PDMS) coated with a permanent magnetic layer (NdFeB) [87]. The proposed architecture made it possible to control two microfluidic channels in parallel, with one valve opening when the other closed, with the applied magnetic force on the polymeric membrane being 8.3 mN, resulting in a membrane displacement of 400 µm.

In the above examples, the actuation principle was based on the fact that a force is applied to the diaphragm which moves when a magnetic field is applied. However, magnetic composites can also be used to make thermally responsive valves: when an alternating magnetic field is applied, the composite heats up, which causes the microvalve to open or close. As show in Figure 11d, Kim et al. [86] developed a system of microvalves based on a ferrowax composite, prepared by mixing paraffin wax with ferrofluid (APG 314). The microvalves, integrated in the microchannels of a lab-on-a-chip disk, were opened through irradiation of an IR laser. The proposed centrifugal microfluidic system was intended for use in an automated diagnostic microsystem involving serial dilution processes.

Other recent examples of heat-sensitive composites for microvalves include the work of Liu et al. on thermally actuated microvalves using paraffin composites by induction heating [88] and Lin et al. on NIR-responsive metal-containing polymer hydrogel for light-controlled microvalves [89].

### 4.4. Magnetic Polymer Composite-Based Micromixers

Micromixers play a key role in the design of drug delivery platforms, biosensing, and integrated lab-on-a-chip for point-of-care applications. The most important features of an effective micromixer are ease of fabrication and integration with other fluidic modules, the ability to handle different solution viscosities, and high mixing efficiency. Micromixers can be classified into two types: passive and active. Passive micromixers rely on the microchannel’s geometry to mix fluids without the need for an external energy source or pressure. On the other hand, active micromixers, which are the primary focus of this study, utilize an external energy source, such as magnetic fields, to actively mix fluids.

**Figure 12:**
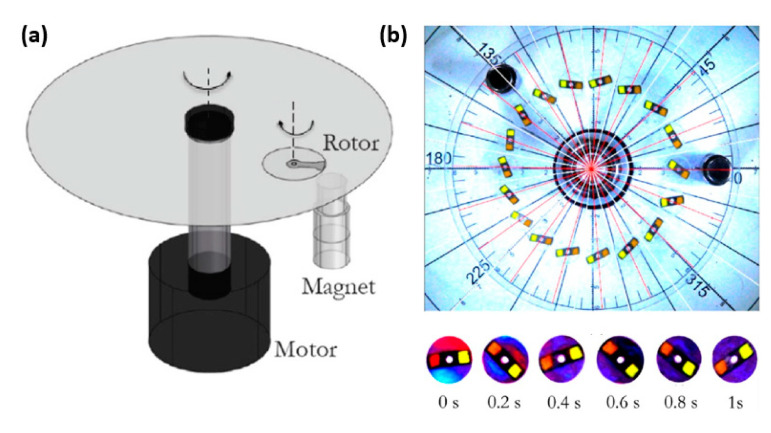
Magnetic polymer composite-based micromixers. On-disk magnetic stirrer for lab-on-a-chip application, (**a**) schematic of the on-disk magnetic stirrer and (**b**) time-lapses of the position of the rotor and magnetically actuated rotor blades during the rotation of the disk at 200 rpm (reproduced with permission from Sensors and Actuators, Elsevier) [90].

Dehghan et al. developed a magnetic stirrer on a rotating microstructured disk used for mixing liquids, which could be applied seamlessly to a wide range of viscosities (up to 42 mPa.s) [90]. The magnetically responsive rotor blades were actuated by a magnet as the disk rotated, allowing the liquid to be mixed (Figure 12a). This integrated microfluidic system aimed at efficient DNA extraction. Dehghan’s design employed steel rotor blades, but there is potential for magnetic stirrer micromixers to be developed in the future using rotor blades composed of magnetic polymer composites.

Current magnetic micromixer research is focused on improving the microscale mixing of ferrofluid and water to create versatile, robust, low-cost, and non-contact microfluidic platforms capable of rapidly mixing fluids for a wide range of biomedical applications, such as biological analysis and diagnosis [91,92,93,94,95,96,97].

### 4.5. Magnetic Polymer Composite-Based Sensors

The unique properties of magnetic polymer composites, such as their mechanical flexibility/elasticity, biocompatibility, and ability to be functionalized to enhance electrical conductivity, in addition to their magnetic properties, make them ideal for use in sensor applications. Furthermore, the polymer matrix allows for straightforward 3D processing using methods such as micro-molding and 3D printing, which facilitates the development of novel sensors with unparalleled properties [98]. An illustration of a sensor based on magnetic polymer composites, potentially used in bio-related applications, is the work of Hu et al. They developed a composite whose resistance varies with an applied external magnetic field. In medical treatments where the human body is exposed to a magnetic field (for example, in hyperthermia treatments with magnetic particles used to heat tissues or organs locally), a locally implanted sensor would make it possible, i.e., to measure in situ and in real time the intensity of the magnetic field in the tissue, in order to avoid overexposure that would lead to overheating of the particles and uncontrolled burns of the cells. Hu et al. developed a conductive magnetorheological elastomer consisting of a conductive polyurethane sponge coated with silver nanowires, embedded in a PDMS matrix itself containing aligned chains of CIP particles. When a magnetic field was applied, a deformation of the matrix was induced by the interaction of the magnetic CIP particle chains, resulting in a variation in the percolation of the silver nanowires. The application of a 428 mT magnetic field resulted in a 200% change in relative resistance of the composite [99].

Likewise, Jang et al. investigated a composite consisting of carbon nanotubes (CNTs) and carbonyl iron (CIP) powder embedded in an Ecoflex (PBTA, polybutylene adipate-co-terphthalate) matrix, resulting in a CIP@CNT cluster-embedded polymeric composite. The percolation threshold for the CNTs was measured to be around 0.75 wt%, and a variation of the resistivity around 10% was measured when a magnetic field of 200 mT was applied [100].

Another example is the magnetic polymer composite developed by Sang et al., combining piezoelectric thermoplastic PVDF (polyvinylidene fluoride) with carbonyl iron. This composite was designed to function as a sensor for both strain and magnetic fields [101]. When subjected to bending deformation, the composite produced piezoelectric charge signals. The measured charges were 3.0 pC and 24.6 pC for 2 mm and 10 mm bending, respectively. Similarly, when subjected to a magnetic field, the composite generated measured charges of 0 pC to 676 pC for applied magnetic fields ranging from 0 to 600 mT. Analogously, another magnetic polymer composite based on PVDF and BiFeO_3_ particles was reported by Pradhan et al. for detection of AC magnetic fields [102].

**Figure 13:**
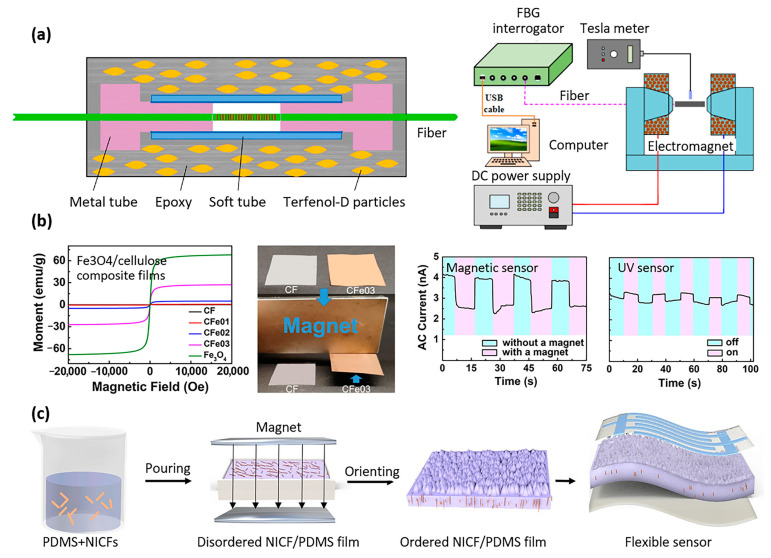
Magnetic polymer composite-based sensors. (**a**) Optical fiber sensor for magnetic field measurement. Schematic configuration of the sensor probe (reproduced with permission from Measurement, Elsevier) [104]. (**b**) Flexible Fe_3_O_4_/cellulose composite film used as magnetic and UV sensor. Left: magnetic hysteresis plots (CF: cellulose film, CFe01-03: Fe_3_O_4_/cellulose composite films). Right: magnetic and UV sensor characteristic (reproduced with permission from Applied Surface Science, Elsevier) [105]. (**c**) Tactile sensor based on magnetically aligned nickel-coated carbon fibers (NICFs) in PDMS, fabrication process and resulting sensor (reproduced with permission from ACS Nano, ACS pub.) [106].

Sensors based on magnetic polymer composites can also be used in biomedical applications to detect rotational movements, e.g., at joints, in the context of rehabilitation after surgery. A 2D rotary sensor, based on a cylinder filled with a magnetic polymer solution, was proposed by Karami et al. [103]. The solution was composed of PDMS dissolved in toluene and nickel microparticles with an average size of 8–20 µm. The cylinder was placed between two fixed and aligned magnets, and a coil was wrapped around this cylinder. The coil was used to detect changes in the magnetic field as the cylinder rotated with respect to the fixed magnets [103]. Beyond biomedical applications, other sensors based on magnetic polymer composites are interesting to mention, as their principles could one day be used in the context of bioMEMS. Peng et al. investigated an optical fiber sensor for magnetic field measurement, based on giant magnetostrictive material (GMM) and fiber Bragg grating (FBG) [104]. The GMM generated magnetostriction in the presence of an externally applied magnetic field, and the FBG was used to detect the magnetostriction. These sensors exhibited a broad measurement range and robust resistance to thermal and mechanical interference, because the magnetostriction of GMM was solely determined by the strength of the applied magnetic field. The architecture of the sensor is illustrated in Figure 13a. The magnetic polymer composite was composed of Terfenol-D particles that were embedded in an epoxy resin matrix. The particles’ orientation was predetermined by applying a magnetic field during the polymerization process. Terfenol-D is an alloy having the highest known magnetostriction properties, expanding and contracting when a magnetic field is applied [104].

Yang et al. demonstrated magnetic and UV sensors made of magnetic composite films consisting of Fe_3_O_4_/cellulose (Figure 13b) [105]. The composites were produced by co-dispersing 0.24 µm-diameter Fe_3_O_4_ particles in cellulose aqueous solution followed by casting. The magnetic sensors functioned through the Fe_3_O_4_ particles’ spin-related scattering in response to an external applied magnetic field, leading to an increase in electrical resistance and a subsequent decrease in current. The UV sensors functioned through the absorption and desorption of oxygen. When exposed to UV illumination, the released and discharged oxygen anions caused by photoelectron reactions interacted with the depletion layer on Fe_3_O_4_, resulting in an enhancement of current intensity [105].

By utilizing the composite magnetic properties, it was possible to establish specific orientations within the material, leading to enhanced sensor properties. For instance, Jiang et al. successfully created a flexible pressure sensor that displayed a sensitivity increase of over two orders of magnitude. This was achieved by incorporating nickel-coated carbon fibers into PDMS and aligning them in the direction of an external magnetic field during the curing process, as depicted in Figure 13c, showing the sensor assembly during manufacturing and upon completion [106]. Wang et al. utilized a comparable approach, presenting a flexible piezoresistive sensor with a sensitive layer made up of a composite material consisting of graphite sheets and nickel nanowires embedded in an EcoFlex polymer matrix. The application of a magnetic field during manufacturing led to the alignment of the fillers in the matrix, resulting in a lower percolation threshold and increased electrical conductivity for the sensor [107].

### 4.6. Other Magnetic Polymer Composite-Based Soft Robots

While the primary focus of this review paper is not on magnetic composites’ use in soft robotics and smart/tunable magneto-actuators, it is worth highlighting the ongoing and thriving research in this area. Soft robotics deals with designing, manufacturing, and controlling robots made from flexible materials rather than rigid components. Developing magneto-active/responsive soft materials is particularly intriguing in this field, as it enables untethered robots to be remotely and wirelessly controlled, allowing for a wide range of complex movements that open up unique opportunities, especially in the biomedical field. Several recent reviews focus on this topic, and Li et al. in particular discuss new materials and structural designs for the engineering of soft robotics and soft actuators with physical intelligence, adaptability, manufacturing scalability and reproducibility, extended lifetime, and end-of-life strategies [108].

A relevant example of medical application is the work of Kim et al., who developed a soft continuum robot that can steer and navigate in any direction for application in the body. The robot could be remotely controlled with magnetic actuation, which involved programming ferromagnetic domains within its soft body. Additionally, a self-lubricating hydrogel layer covered the surface of the robot to enhance its locomotion performance [109].

Different innovative approaches have been developed to give specific local orientations to the magnetic particles in the composites, allowing the development of soft robots and actuators with the desired shapes and properties. Alapan et al. proposed a flexible technique to encode reprogrammable shape-morphing instructions into magnetic soft machines through a high-throughput process [110]. This approach involved heating the magnetic composites above their Curie temperature and reorienting their magnetic domains with external magnetic fields during cooling. They demonstrated discrete, 3D, and reprogrammable magnetization with spatial resolution as high as ~38 μm, and high throughput using the contact transfer of distributed magnetization profiles from a magnetic master (~10 samples/min using a single master) [110].

In a different approach, Song et al. developed a magnetic composite based on magnetic microspheres in an elastomeric matrix, where the magnetic microspheres were composed of ferromagnetic microparticles encapsulated with oligomeric-PEG. By controlling the encapsulating polymer phase transition, the magnetization profiles of the magnetic composite could be rewritten by physically realigning the ferromagnetic particles [111].

The versatile fabrication of complex 3D structures from a single platform and single composite formulation is essential to enable magnetic soft robots and actuators to reach the general public. Xu et al. described a method that employs UV lithography to encode 3D magnetization at the submillimeter scale in planar flexible composites. Their approach involved reorienting pre-magnetized permanent magnetic particles and then selectively curing UV resin to create patterns of local magnetization. With this method, the team was able to fabricate multiple microrobots with distinct geometries and 3D magnetization profiles using a single precursor in a single process, achieving a geometrical feature size as small as 100 × 100 μm and a precise magnetization feature size as small as 250 × 250 μm on an 80-μm-thick UV resin layer [112]. In addition, as highlighted in Section 3, 3D printing has emerged as a crucial manufacturing method for the production of flexible robots and actuators that employ soft magnetic composites. This approach has been extensively studied in the field, demonstrating its potential for enabling the fabrication of complex devices with intricate geometries and unique functionalities [113,114,115,116,117,118,119,120].

Furthermore, when it comes to actuators, it is not just the initial shape and resulting 3D deformation that are crucial but also the force generated by the device. This aspect has been explored, e.g., by Schmauch et al., who fabricated actuators based on polyurethane elastomer films filled with carbonyl iron microparticles aligned in a programmed direction. The manufactured cantilevers generated a specific torque as high as 68 Nm/kgT for applied magnetic fields up to 0.6 T, enabling a 2.6 cm cantilever to lift 50 times its own weight [121].

## 5. Discussion

In recent years, various approaches have been employed in the development of magnetic polymer composites, particularly in the selection of magnetic particles. Superparamagnetic iron oxide (Fe_3_O_4_) nanoparticles (SPIONS) have been utilized due to their desirable magnetic properties and ease of preparation, resulting in homogeneous composites. Due to their nanoscale dimensions, these particles consist of a single magnetic domain that constantly fluctuates at room temperature and possesses negligible remanent magnetization. Consequently, these magnetic particles do not exhibit attractive or repulsive behavior in the absence of a magnetic field, making them easier to mix in viscous solutions during the production of magnetic polymer composites. Larger Fe_3_O_4_ magnetic particles with a diameter in the micrometer range have also been utilized due to their ability to achieve greater magnetization. Furthermore, the larger particle size simplifies the handling process during composite mixing, as health-related safety concerns necessitate that nanometer-sized powders be handled in glove boxes to avoid hazardous inhalation for the preparers [19,21,22,23,24,27,28,29,30,31,32,33,34,35,36,37,38,39,40,41,44,45,46,48,50,57,58,59,60,61,63]. Alternatively, iron or carbonyl iron particles (CIP) have been used for their high magnetization [42,43,47], while other researchers have opted for ground neodymium–iron–boron magnet (NdFeB) particles, which possess the highest magnetization but present greater challenges during preparation. A challenge in the preparation of NdFeB particles is in the permanent magnet nature of the material made from an alloy of neodymium, iron, and boron. During the grinding process, the particles attract each other and form clusters that are difficult to separate, making it challenging to obtain powders with a homogeneous particle size [17,20,40,49]. These latter composites are more suited for soft robotics applications or uses outside the human body, as rare-earth permanent magnets and their powder are not biocompatible or biodegradable, unlike iron and iron oxide particles. This is one reason why all biodegradable composites have used iron oxide particles (Table 4). A special case is the work of Charlet et al. To prepare PEG hydrogels, they mixed PEG with an aqueous solution of Fe^3+^ and Fe^2+^ ions, which led to gelation, and imparted magnetic properties to the hydrogels [18]. Shibaev et al. and Zhao et al. employed CoFe_2_O_4_ and ZnFe_2_O_4_, respectively, for the preparation of their self-healing composites [25,26].

Self-healing, shape memory, and biodegradability are important properties that can significantly improve the functionality of microactuators, micropumps, and other microsystems, especially in the medical field. Self-healing materials are highly desirable in bioMEMS devices since they can increase the lifespan and safety of key components, which is critical for medical implants. Shape-memory materials can also be very useful in microactuators, micromanipulators, and medical catheter guides, where remote and predefined shape changes are required for proper functionality and in order to, e.g., manipulate tissues in difficult to access areas and in the context of minimal invasive surgery. Biodegradability is particularly essential in bioMEMS designed for medical implants that are only necessary for a short period, as they eliminate the need for surgical removal. All these properties offer new possibilities and have the potential to significantly improve the reliability and safety of the new generation of bioMEMS devices.

A diverse range of polymers and hydrogels have been used for the matrix of the composites, including self-healing (Table 2), shape-memory (Table 3), and biodegradable (Table 4) magnetic polymer composites, resulting in a wide variety of mechanical and chemical properties. On the other hand, detailed observation of recent advances in biomedical MEMS based on magnetic polymer composites shows that, except for drug delivery applications, most other microsystems such as microactuators (Section 4.1 and Figure 8 and Figure 9), micropumps (Section 4.2 and Figure 10), microvalves (Section 4.3 and Figure 11), and micromixers (Section 4.4 and Figure 12) are based solely on PDMS mixed with Fe_3_O_4_ or NdFeB particles. Sensors, on the other hand, present a greater variety of substrates, depending on the sensing mechanism and the intended application. For example, Yang et al. fabricated magnetic and UV sensors on cellulose substrate [105], and Wang et al. used EcoFlex for their piezoresistive sensor [107].

Establishing common figures of merit will be crucial for advancing the research in magnetic polymer composites and accurately quantifying their self-healing, shape-memory, and biodegradability properties. This will facilitate better comparison and selection of the most promising composite families for future commercial bioMEMS applications. The field therefore needs to mature by defining these common metrics to better focus research efforts and accelerate the development of new and improved magnetic polymer composites.

This review highlights that the potential of recently developed magnetic polymer composites has yet to be fully realized in the development of the latest biomedical MEMS utilizing electromagnetic actuation. This presents a technological gap that, if addressed, has the potential to yield much more efficient microsystems for the biomedical field in the future. For instance, the incorporation of self-healing, shape-memory, and biodegradable materials as building blocks for biomedical implants could enhance device safety (by enabling self-repair), functionality (by allowing implants to adapt to changing needs), and patient comfort (by eliminating the need for a second surgery for device removal in the context of short-use implants). The incorporation of these novel composite materials in the production of upcoming biomedical MEMS requires the development of process flows that are compatible with microfabrication techniques in cleanroom environments. This is crucial to enable the structuring of these materials at the micro-scale and their integration with conventional integrated circuits and MEMS components.

## 6. Conclusions

As a conclusion, the objective of this review was to investigate the potential of functionalized magnetic polymer composites’ use in electromagnetic micro-electro-mechanical systems (MEMS) for biomedical applications. The review first examined recent advancements in magnetic polymer composites that possess unique features such as self-healing capabilities, shape-memory, and biodegradability. The analysis conducted revealed the predominant utilization of three distinct categories of magnetic particles, namely Fe_3_O_4_, Fe/CIP, and NdFeB, in conjunction with a diverse range of polymers and hydrogels, evaluated for their chemical and mechanical characteristics, as well as their potential for self-healing, shape-memory, and biodegradability. Subsequently, the review focused on electromagnetic MEMS for biomedical applications, including microactuators, micropumps, miniaturized drug delivery systems, microvalves, micromixers, and sensors. The identified technological gap between recently developed functionalized magnetic polymer composites and the materials currently used in electromagnetic biomedical MEMS presents an opportunity to enhance the efficiency of microsystems in the biomedical field through the incorporation of self-healing, shape-memory, and biodegradable materials. These materials have the potential to improve device safety, functionality, and patient comfort. However, the integration of these materials in bioMEMS requires the development of process flows that are compatible with conventional IC and MEMS microfabrication techniques in cleanroom environments.

## Figures and Tables

**Table 1:** Comparison of the properties of magnetic polymer composites versus non-magnetic polymer composites for bioMEMS applications.

Properties	Description	Non-Magnetic Polymer Composites	Magnetic Polymer Composites
Biocompatibility	No adverse reactions observed when the polymer composite comes into contact with biological tissue	Yes	Yes
Tailored properties	Polymer composites designed to have specific chemical, mechanical, electrical, and thermal properties	Yes	Yes
Lightweight	Lightweight and thin bioMEMS are important for medical devices that need to be small and portable	Yes	Yes
Easy processing	Polymer composites can be easily processed into complex shapes and structures using a variety of techniques (3D printing, molding, cleanroom microfabrication)	Yes	Yes
Magnetic properties at the macroscopic scale	Applying an external magnetic field on a device made of magnetic polymer composite (e.g., membrane, cantilever) allows (1) attracting the object by the generated magnetic force, (2) deforming/influencing the orientation of the object by the applied magnetic torque, with various applications in bioMEMS	No	Yes
Magnetic properties at the bulk material scale	Applying an external magnetic field on a magnetic polymer composite makes it possible to change the internal structure of the material, influencing: - the chemical properties (making it possible to obtain, e.g., specific self-healing, shape-memory properties) - the mechanical properties (reinforcing the mechanical strength along a certain axis) - the electrical properties (resulting, e.g., in increased conductivity in magnetic composites loaded with conducting particles)	No	Yes
Applications in bioMEMS	Polymer composites are of particular interest for all applications that require devices that are mechanically compliant with cells or with the various organs of the human body. In addition, magnetic polymer composites allow for all bioMEMS applications in which an interaction with an external magnetic field allows for the control of, e.g., drug delivery devices, sensors, and actuators.		

**Table 2:** Self-healing magnetic polymer composites, mechanism, composition performance, and applications.

Self-Healing Mechanism	Matrix	Filler	Input	Output	Application	Ref.
Via direct mixing						
Disulfide bonds	Polymerized alpha lipoic acid (ALA)	NdFeB	Magnetic Field	Actuation	Soft robotics	[17]
(Metal) ion bond	Functionalized PEG hydrogel with pyrogallols	Fe/Ca/Zn	Autonomous/close distance	Fast self-healing adhesive	Biocompatible wound sealant	[18]
Intermolecular diffusion	Ethylene glycol	Fe_3_O_4_	Heat or magnetic field	Phase transformation self-healing	Cell engineering	[19]
Diels–Alder	Polyethertriamine Jeffamine	Fe_3_O_4_	Magnetic field and heat	Closure of large gaps	Soft robotics	[21]
Dipole-interaction	Dowsil CY52-276 gel	NdFeB and CIP	Autonomous/residual magnetization	Instantaneous self-healing regardless of cycles	Bioengineering and soft robotics	[20]
Covalent bonds	Chitosan	Fe_3_O_4_	Magnetic field	Actuation while shape shifting	Biocompatible injectable	[22]
Schiff base	Chitosan/alginate hydrogel and gelatin	Fe_3_O_4_	Magnetic field	Self-healing under physiological conditions	Drug delivery	[23]
Metal ligand complex	Dopamine-functionalized polyurethane	Fe_3_O_4_ with MoS_2_	Heat and shape-memory	EMI shielding	Stretchable electronics	[24]
Ionic bond (disulfide, hydrogen, iron–carboxylate)	Thioctic acid	Fe_3_O_4_	Autonomous	100% healing efficiency	Shape construction	[27]
Hydrogen bond	Carboxymethyl hydroxypropyl guar	CoFe_2_O_4_	Magnetic field	100% healing efficiency and actuation	Soft robotics	[25]
Boric acid ester and hydrogen bonds	Polypyrrole PPy	ZnFe_2_O_4_ and MWCNT	Autonomous	Self-healing	Electronic skins and drug delivery	[26]
Via surface engineering						
Hydrogen bonds	NVP-DVB	Fe_3_O_4_	Magnetic field	Self-healing and weight bearing	Soft robotics	[28]
Via additive manufacturing						
Schiff base	Polysaccharides	Fe_3_O_4_	Magnetic field	Printed structure alteration	3D tissue engineering	[29]
Via self-assembly						
Metal–ligand complex	Polyurethane	Fe_3_O_4_	Autonomous	Fast and efficient self-healing during actuation	Smart biomimetic devices	[30]

**Table 3:** Shape-memory magnetic polymer composites, composition performance, and applications.

Matrix	Filler	Input	Output	Application	Ref.
Via solvent casting					
Amorphous acrylate-based polymers	Fe_3_O_4_ and NdFeB particles	AC magnetic field	Carry a weight 64x heavier than own weight	Autonomous soft robots, minimally invasive surgery, digital logic circuits	[40]
Bio-based benzoxazine resin	Fe_3_O_4_ particles	AC magnetic field Light	Highest shape recovery of 99% within 26 s for 5 wt% Fe_3_O_4_	Soft robotics	[46]
Poly(ε-caprolactone) (PCL)	Fe_3_O_4_ particle	AC magnetic field Light Direct heating	Fixity ratio >99%, recovery ratio >95%	Soft robotics	[41]
Epoxy resin (EP)	Fe particles	AC magnetic field Near-infrared	Microconed surface with anisotropic droplet sliding ability	Surfaces with tunable wettability, liquid transport, temperature switch	[42]
Thermoplastic polyurethane	Carbonyl iron particles	AC magnetic field Light	Recovery of permanent shape of the cilia after 30 s	Biomedical applications, microrobot	[43]
Thermoplastic polyurethane	Fe particles	AC magnetic field Light	Shape recovery >95.5% Excellent biocompatibility in vitro	Reconfigurable optics, surfaces with tunable wettability	[47]
Polylactic acid (PLA)	Fe_3_O_4_ particle	AC magnetic field	Snapper transition extremely sharp (>90%)	Scaffolds for bone repair	[44]
Carboxylic styrene-butadiene rubber (XSBR)	Fe_3_O_4_ particle, zinc dimetha-crylate (ZDMA)	AC magnetic field Heat	Fixity ratio >99%, recovery ratio >99%, tensile strength reached >30 MPa	Biomedical applications	[45]
Poly(ε-caprolactone) (PCL), thermoplastic polyurethane (TPU)	Fe_3_O_4_ particle, polydopamine	AC magnetic field Light	Fast light- and magnetic-responsive ability (magnetic field: 1 s; light: 5 s)	Soft robotics, artificial muscles	[48]
Via 3D printing					
Acrylate-based amorphous polymers	NdFeB particle	AC magnetic field Heat	Surfaces with controllable varying Poisson’s ratio	Soft robotic, biomedical applications	[49]
Polylactic acid (PLA)	Fe_3_O_4_ particle	AC magnetic field Heat	Fe_3_O_4_ enhances the mechanical strength of PLA by 17%	Personalized bio-designed tracheal scaffolds	[50]
Polylactic acid (PLA)	Fe_3_O_4_ particle	AC magnetic field Heat	Shape recovery in few seconds. Shape recovery at 40 °C, relevant in vivo	Bone tissue repair, biomedical applications	[51]

**Table 4:** Biodegradable magnetic polymer composites, composition performance, and applications.

Matrix	Filler	Fabrication	Output	Application	Ref.
Polylactic acid (PLA)	Fe_3_O_4_ particles	Extrusion 3D printing	PLA is biodegradable within up to two years	Scaffolds for bone repair, tracheal scaffolds	[44,50,51]
Poly(ε-caprolactone), polyurethane	Fe_3_O_4_ particles, polydopamine	Solvent casting	PCL is biodegradable within up to two years	Soft robotics, artificial muscles	[48]
Gelatin methacryloyl	Fe_3_O_4_ particles	Two-photon polymerization	Complete degradation after two weeks	Helicoidal microrobots for drug delivery	[57,58]
Poly(ethylene glycol) diacrylate (PEGDA), pentaerythritol triacrylate (PETA)	Fe_3_O_4_ particles, 5-fluorou- racil	Micro machining + dip coating	Drug release from the microrobots investigated over 6 weeks	Multi-shape microrobots for drug delivery	[59,63]
Calcium alginate hydrogel (Ca-Alg)	Fe_3_O_4_ particles	Hydrodynamic electrospray ionization jetting	Ultrasound actuation used for on-demand drug release	Loaded capsules for drug delivery	[60]
Methacrylamide Chitosan (chMA)	Fe_3_O_4_ particles	Two-photon polymerization	Delivery of drugs and other cargo (gene, stem cell, imaging agent)	Helicoidal microrobots for drug delivery	[61]

## Data Availability

Data are available on request to C.M.B.
